# Synergistic Reinforcement and Multimodal Self-Sensing Properties of Hybrid Fiber-Reinforced Glass Sand ECC at Elevated Temperatures

**DOI:** 10.3390/polym18030322

**Published:** 2026-01-25

**Authors:** Lijun Ma, Meng Sun, Mingxuan Sun, Yunlong Zhang, Mo Liu

**Affiliations:** 1 School of Transportation Science and Engineering, Jilin Jianzhu University, Changchun 130118, China; malijun@jlju.edu.cn (L.M.); 15545342138@163.com (M.S.); 2022019007@ccccltd.cn (M.S.); 2 School of Transportation and Civil Engineering & Architecture, Foshan University, Foshan 528225, China

**Keywords:** glass aggregate, polypropylene fiber, carbon fiber, high-temperature resistance, resistivity, mechanical properties, self-sensing properties, microstructure

## Abstract

To address the susceptibility of traditional concrete to explosive spalling and the lack of in situ damage-monitoring methods at high temperatures, in this study, a novel self-sensing, high-temperature-resistant Engineered Cementitious Composite (ECC) was developed. The matrix contains eco-friendly glass sand reinforced with a hybrid system of polypropylene fibers (PPFs) and carbon fibers (CFs). The evolution of mechanical properties and the multimodal self-sensing characteristics of the ECC were systematically investigated following thermal treatment from 20 °C to 800 °C. The results indicate that the hybrid system exhibits a significant synergistic effect: through PFFs’ pore-forming mechanism, internal vapor pressure is effectively released to mitigate spalling, while CFs provide residual strength compensation. Mechanically, the compressive strength increased by 51.32% (0.9% CF + 1.0% PPF) at 400 °C compared to ambient temperature, attributed to high-temperature-activated secondary hydration. Regarding self-sensing, the composite containing 1.1% CF and 1.5% PPF displayed superior thermosensitivity during heating (resistivity reduction of 49.1%), indicating potential for early fire warnings. Notably, pressure sensitivity was enhanced after high-temperature exposure, with the 0.7% CF + 0.5% PPF group achieving a Fractional Change in Resistivity of 31.1% at 600 °C. Conversely, flexural sensitivity presented a “thermally induced attenuation effect” primarily attributed to high-temperature-induced interfacial weakening. This study confirms that the “pore-formation” mechanism, combined with the reconstruction of the conductive network, governs the material’s macroscopic properties, providing a theoretical basis for green, intelligent, and fire-safe infrastructure.

## 1. Introduction

With the rapid expansion of the global infrastructure, the engineering sector faces increasingly stringent demands regarding the sustainability and structural safety of construction materials. Although widely used, ordinary concrete suffers from inherent brittleness, making it highly susceptible to cracking under complex loading or harsh environmental conditions. This deficiency severely compromises the long-term durability and service safety of structures [1]. To overcome these limitations, Engineered Cementitious Composites (ECCs) have emerged as high-performance ductile materials. Based on their micromechanical design principles, ECCs exhibit superior tensile strain-hardening behavior and crack control capabilities, demonstrating immense potential for applications in structural repair, seismic retrofitting, and large-scale infrastructure projects [2,3,4,5,6].

In recent years, enhancing the sustainability and lowering the carbon footprint of ECCs has become a focal point of research. One core strategy involves identifying alternative binders and aggregates [7]. Eco-friendly glass sand (GS), a widely available and low-cost recycled waste resource, is regarded as an ideal alternative to natural silica sand due to its stable physicochemical properties [8]. Replacing silica sand with GS when preparing ECCs not only effectively addresses the challenges of waste glass disposal but also significantly lowers the carbon footprint of production. Studies indicate that incorporating GS can reduce CO_2_ emissions by approximately 27.5 kg/m^3^ while synergistically enhancing the compressive, tensile, and flexural strengths of ECCs [9], thereby providing a viable pathway for developing green, high-performance ECCs.

While traditional Engineered Cementitious Composites (ECCs), typified by Polyvinyl Alcohol (PVA) fibers, exhibit superior ductility and crack control capabilities at ambient temperatures, there are severe challenges to improving their performance under elevated temperatures, thereby restricting their application in extreme thermal environments. The polymer fibers widely employed in current ECC formulations (such as PP, PVA, and PE) possess limited thermal stability [10,11,12], with melting points typically ranging only from 170 °C to 280 °C [13,14,15,16]. Exposure to high temperatures induces rapid fiber melting, leading to the failure of bridging and crack-arresting mechanisms, which directly results in a significant degradation in residual tensile strength [17,18]. Furthermore, elevated temperatures accelerate the decomposition of matrix hydration products (e.g., C-S-H, CH), further weakening the microstructural framework of the material [19,20]. However, this ‘fiber melting’ phenomenon plays a pivotal role in preventing catastrophic failure. Unlike ordinary concrete, which is prone to explosive spalling within the range of 250–400 °C [21], the melting of polymer fibers within the ECC matrix creates an interconnected network of micro-channels. These channels function as ‘vapor pressure relief valves,’ effectively facilitating the escape of high-pressure water vapor generated at elevated temperatures, thereby alleviating internal thermal stress and mitigating spalling [22,23,24,25,26,27,28]. This characteristic trade-off—between partial mechanical strength and structural integrity and spalling resistance—holds significant importance for the collapse resistance of structures during fire incidents [29].

Given the concealed nature of fire-induced damage, establishing an effective Structural Health Monitoring (SHM) system is vital for assessing post-disaster safety. Traditional external sensors suffer from inherent deficiencies regarding compatibility with the concrete matrix and high-temperature durability [30]. Leveraging the excellent crack control capabilities of ECCs, the incorporation of functional fillers to endow the material with self-sensing properties represents a new frontier for intelligent in situ monitoring. Currently, progress has been made in research involving conductive fillers such as carbon fibers (CFs) and carbon black (CB) [31,32,33,34]. Among these, CFs have been proven to significantly enhance piezoresistive sensitivity due to their high aspect ratio, which facilitates the formation of conductive networks [35]. Crucially, incorporating carbon fibers (CFs) to construct a hybrid fiber system effectively reconciles the conflict between ‘strength retention’ and ‘spalling resistance’ inherent in single polymer fiber-reinforced systems at elevated temperatures. CFs possess excellent high-temperature resistance, effectively inhibiting microcrack propagation and increasing the cracking threshold within the 200–800 °C range [36]. Previous studies have found that Reactive Powder Concrete (RPC) incorporating CFs exhibits a significantly higher residual mechanical strength after high-temperature exposure compared to plain control groups [37,38], suggesting a potential for performance improvements in ECCs under high-temperature conditions.

In summary, although studies exist assessing glass sand ECCs, high-temperature-resistant ECCs, and self-sensing concrete independently, the integration of these three domains to develop a novel composite that combines environmental sustainability, high-temperature spalling resistance, and in situ self-sensing capabilities remains an unexplored frontier. Theoretically, the “pore-formation and pressure-relief” mechanism of PPFs and the “high-temperature conductivity” of CFs offer excellent complementarity. However, the hybrid effect of these two fibers within a glass sand ECC matrix, as well as their evolution throughout the heating process, remains unclear. Consequently, in this study, eco-friendly glass sand is utilized to replace traditional silica sand to develop a novel self-sensing, high-temperature-resistant ECC using different ratios of PP and CF. The residual mechanical properties and multi-functional self-sensing characteristics of the material were systematically investigated following thermal treatment ranging from 20 °C to 800 °C. Furthermore, microstructural analysis was performed to elucidate the material’s damage evolution mechanisms, aiming to provide a theoretical basis for the development of the next generation of green, intelligent civil engineering materials.

## 2. Materials and Methods

### 2.1. Test Materials

**Figure 1:**
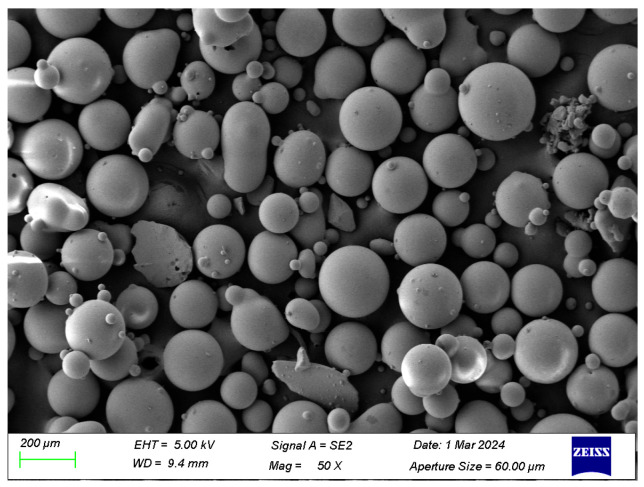
Grain structure of glass sand under SEM.

**Figure 2:**
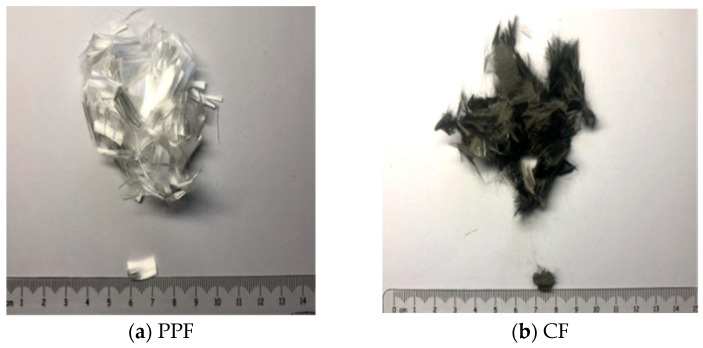
Fiber sizes and styles.

The primary binder used in this study was P·II 42.5 Portland cement produced by Jilin Yatai Cement Co., Ltd. (Changchun, China), which conforms to the Chinese standard Common Portland Cement (GB 175-2007) [39]. Its specific technical parameters are listed in Table 1. Silica fume and fly ash, serving as supplementary cementitious materials, were provided by Hebei Shengyi Mineral Products Trading Co., Ltd. (Shijiazhuang, China). Both materials comply with the Technical Code for Application of Mineral Admixtures (GB/T 51003-2014) [40]; their relevant parameters are detailed in Table 2 and Table 3, respectively. For the fine aggregate, 100-mesh glass sand (GS) with a particle diameter of 0.15 mm was employed, sourced from Hongyang Cleaning Equipment Co., Ltd. (Ningbo, China). The chemical composition of the GS is presented in Table 4, and its morphological characteristics, as observed under Scanning Electron Microscopy (SEM), are shown in Figure 1. The hybrid fiber system consisted of polypropylene (PP) fibers and carbon fibers (CFs). The PPFs (12 mm in length) were manufactured by Beijing Zhongfang Fiber Construction Technology Co., Ltd. (Beijing, China), while the CFs (6 mm in length) were produced by Shanghai Lishuo Composite Material Technology Co., Ltd. (Shanghai, China). Detailed specifications and performance parameters for both fiber types are provided in Table 5 and Table 6, and their physical appearances are displayed in Figure 2. To ensure workability and fiber dispersion, chemical admixtures were utilized. A polycarboxylate ether-based high-performance superplasticizer produced by Shanxi Feike New Material Technology Co., Ltd. (Yuncheng, China) was used to adjust the rheology; its physical and chemical properties are summarized in Table 7. Additionally, Sodium Carboxymethyl Cellulose (CMC-Na) with a viscosity of 300 mPa·s, produced by Fuchen Chemical Reagent Co., Ltd. (Tianjin, China), was added as a dispersant. Tributyl phosphate, manufactured by Wuxi Yatai United Chemical Co., Ltd. (Wuxi, China), served as the defoamer, with an acidity (calculated as H+) of mmol/10 g ≤ 0.2%.

### 2.2. Mixing Ratio Design

To systematically investigate the effects of varying volume fractions of PPFs and CFs on the mechanical and self-sensing performance of the ECC, a total of 10 mix proportions were prepared, as detailed in Table 8. The experimental variables included three PPF volume fractions (0.5%vol., 1.0%vol., 1.5%vol.) and three CF volume fractions (0.7%vol., 0.9%vol., 1.1%vol.). A fiber-free mixture was also prepared as the control group. To optimize the matrix performance, three chemical admixtures were incorporated into all mixtures, with their dosages calculated by the weight of the binder materials. Specifically, 0.035% Sodium Carboxymethyl Cellulose (CMC) was added as a dispersant to facilitate a uniform fiber distribution within the matrix. To minimize porosity, caused by air entrapment, and thereby densify the matrix structure, 0.15% defoamer was introduced. Additionally, a high-efficiency superplasticizer (1.2%) was utilized to ensure adequate workability of the fresh mixture.

### 2.3. Test Methods

To evaluate the residual mechanical, electrical, and self-sensing performance of the glass sand-based self-sensing ECC following exposure to various elevated temperatures, five target temperature levels were selected: 20 °C (serving as the ambient control), 200 °C, 400 °C, 600 °C, and 800 °C. Heating was conducted as follows: The specimens were heated to the designated target temperatures at a heating rate of 10 °C/min. This temperature was maintained for 2 h to ensure thermal equilibrium within the specimens. Finally, the specimens were allowed to cool naturally to room temperature for 24 h prior to subsequent performance testing.

#### 2.3.1. Mechanical Performance Testing

The fundamental mechanical properties of the specimens were evaluated in accordance with the Standard for Test Methods of Concrete Physical and Mechanical Properties (GB/T 50081-2019) [41]. Specifically, for compressive strength and splitting tensile strength tests, cubic specimens with dimensions of 100 mm × 100 mm × 100 mm were utilized. The loading rates were set at 0.5 MPa/s and 0.05 MPa/s for each test, respectively. In addition, all mechanical tests (compressive, splitting tensile, and flexural) were conducted using three replicate specimens to ensure data accuracy. Given that the plate specimens underwent severe matrix deterioration and became highly brittle after exposure to temperatures above 600 °C, the gripping pressure in traditional uniaxial tensile tests would inevitably cause stress concentration, leading to premature failure outside the gauge length. Therefore, the four-point bending test was adopted to evaluate the material’s flexural toughness and deformability by examining the deflection-hardening behavior presented in the load–deflection curves. Flexural strength was determined using prismatic specimens measuring 400 mm × 100 mm × 100 mm, with a constant loading rate of 0.05 MPa/s. The flexural toughness test was conducted in reference to the Test Methods for Properties of Glass Fiber Reinforced Cement (GB/T 15231-2008) [42]. The specimens for this test were prepared as thin plates measuring 400 mm × 100 mm × 15 mm. Unlike the force-controlled methods used above, a displacement-controlled loading mode at a rate of 0.1 mm/min was employed in this test to accurately capture the post-peak deformation behavior.

#### 2.3.2. Self-Sensing Performance Testing

Under the application of a constant DC voltage, current readings in concrete often exhibit decay and fluctuation due to ion migration and polarization effects within the matrix. To eliminate signal drift caused by polarization, a low-voltage pre-energization strategy was adopted. A DC voltage below 2 V was applied to prevent water electrolysis, and the circuit was energized continuously until the current reached a steady state. Current data were acquired using a digital multimeter (VICTOR 890D) at a sampling interval of 5 s. The volume resistivity and Fractional Change in Resistivity (FCR) were calculated using Equation (1) and Equation (2), respectively.

**Figure 3:**
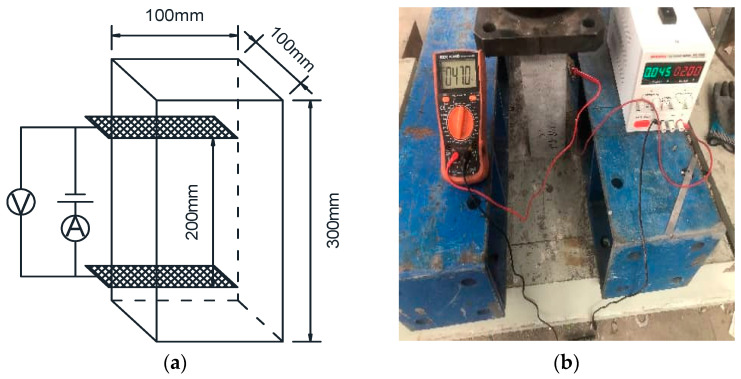
Pressure sensitivity testing. (**a**) Arrangement of pressure-sensitive electrodes. (**b**) Loading method.

The pressure sensitivity under compression was evaluated using prismatic specimens measuring 300 mm × 100 mm × 100 mm. Copper mesh electrodes (60 mm × 80 mm) were symmetrically embedded within the concrete at a depth of 40 mm, with a spacing of 200 mm (Figure 3a). The test employed a force-controlled cyclic loading mode at a rate of 1 kN/s (Figure 3b). Prior to formal testing, multiple pre-loading cycles were conducted to close initial micro-cracks, eliminate mechanical slack, and ensure the specimen operated within the elastic stage.

**Figure 4:**
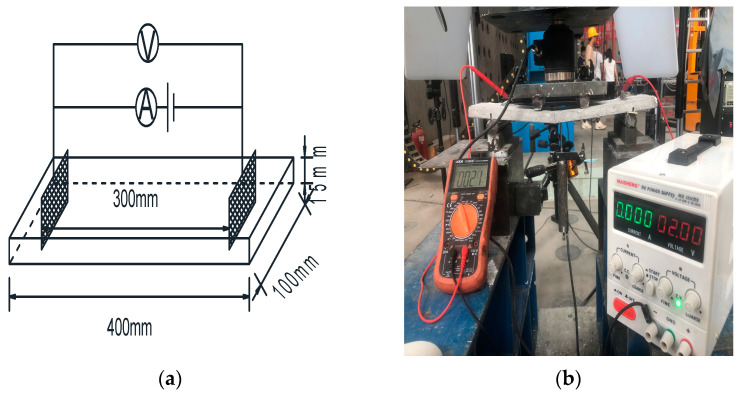
Bending sensitivity measurement. (**a**) Arrangement of bending-sensitive electrodes. (**b**) Loading method.

**Figure 5:**
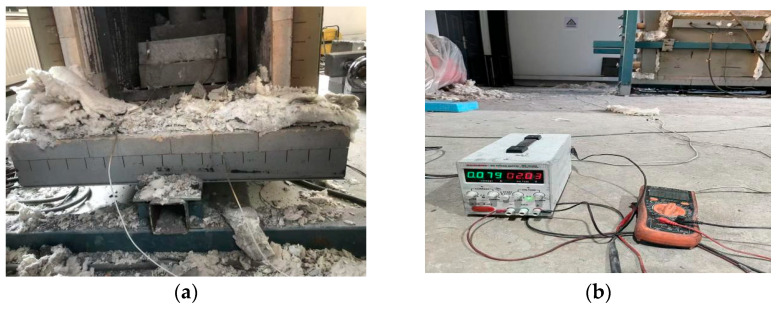
Temperature sensitivity test. (**a**) Heating environment and specimen placement; (**b**) Electrical resistance measurement configuration.

The flexural sensitivity test utilized a monotonic loading mode at a rate of 0.1 mm/min. The specimens were thin plates (400 mm × 100 mm × 15 mm) with embedded copper mesh electrodes (40 mm × 15 mm) spaced 300 mm apart (Figure 4a). A load cell and a displacement transducer were arranged to synchronously record the load–displacement curves (Figure 4b). Strict experimental protocols were adopted to guarantee the reliability and reproducibility of the sensitivity tests at 800 °C. To minimize physical interference, large specimens (400 mm × 100 mm × 100 mm) were used to increase thermal inertia and ensure a uniform internal temperature distribution, while high-temperature mica-insulated wires were utilized to prevent oxidation-induced resistance errors. For data accuracy, resistance measurements were taken solely upon reaching thermal equilibrium at each target temperature. The robustness of the findings was further validated through triplicate tests for each mix proportion, confirming the accuracy of the sensing signals up to 800 °C (Figure 5).

(1) $$\rho =\frac{US}{IL}$$

where

ρ—resistivity, Ω·cm;
*U*—specimen two electrode terminal voltage, V;
*S*—specimen cross-sectional area, cm^2^;
*I*—electric current, A;
*L*—electrode distance, cm.

(2) $$FCR=\frac{\rho -\rho_{0}}{\rho_{0}}\times 100\%$$

where

*FCR*—Fractional Change in Resistivity, %;
ρ—resistivity, Ω·cm;
$\rho_{0}$—initial resistivity, Ω·cm.

## 3. Results and Discussion

### 3.1. Physical Observations

**Figure 6:**
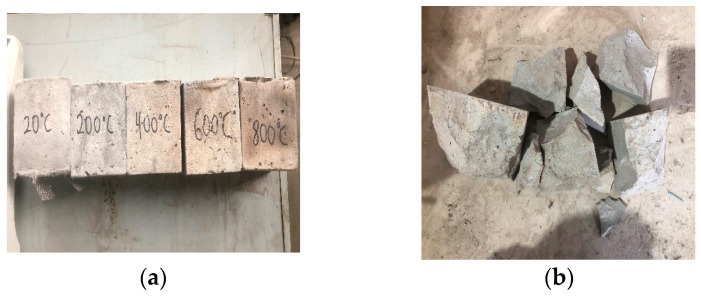
Failure patterns of the specimens: (**a**) fiber-reinforced concrete; (**b**) plain concrete.

The experiments outlined in this section assess spalling resistance primarily through macroscopic visual inspections, serving as a proof-of-concept verification for the suppression of thermally induced explosive spalling through the use of the hybrid fiber system. Figure 6a presents the macroscopic surface morphology of the ECC specimens subjected to various temperatures. Overall, the hybrid fiber-reinforced ECC exhibited exceptional structural integrity under high-temperature conditions. Notably, even after exposure to 800 °C, no significant macroscopic penetrating cracks were observed on the specimens’ surfaces. However, as the temperature increased, the matrix underwent distinct color evolution and deterioration in surface texture.

Specifically, at ambient temperature (20 °C), the specimen surface presented a uniform light gray appearance. Upon heating to 200 °C, the color shifted to dark gray, accompanied by a slight increase in visible surface porosity. At 400 °C, the matrix hue transitioned to light yellow, and the pore aperture expanded significantly. As the temperature reached 600 °C, localized gray-black areas emerged and scale-like precursors (resembling fish scales) were observed, indicating that high temperatures had induced initial microstructural damage. By 800 °C, the specimens had turned deep black overall; distinct delamination and spalling occurred at the edges and the surface pore structure was further coarsened. Notably, the ECC groups containing PPFs exhibited exceptional thermal stability, with no explosive spalling occurring throughout the entire temperature range. This performance is attributed to the “pore pressure relief mechanism”: at high temperatures, the PPFs melt and form an interconnected network of micro-channels within the matrix. These channels effectively act as release valves for the internal steam pressure generated by moisture vaporization, thereby preventing the internal tensile stress from exceeding the tensile strength of the matrix. Conversely, the fiber-free control group suffered catastrophic explosive spalling at 400 °C (Figure 6b), confirming that a plain matrix is incapable of resisting tensile failure induced by high-pressure steam.

**Figure 7:**
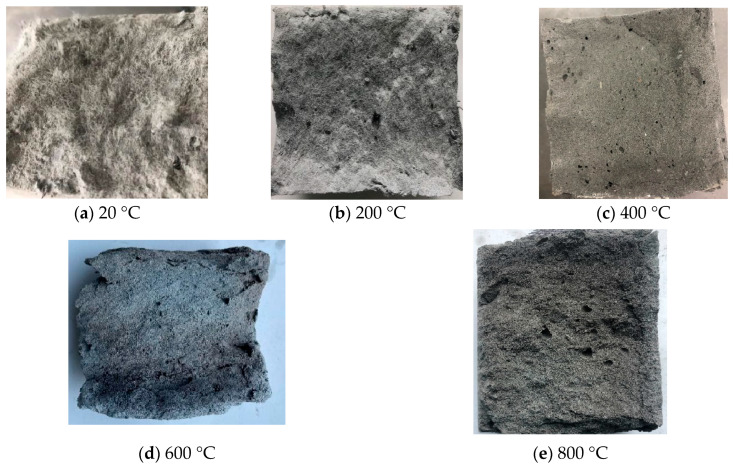
Internal state of concrete after high-temperature testing.

Figure 7 reveals the evolution of the internal cross-sectional state of the ECC at different temperatures. In the 20 °C to 200 °C range, initial melting of the near-surface PPFs was observed. As the temperature rose to 400 °C, extensive melting of the internal PPFs occurred, accompanied by the escape of volatile smoke, leaving behind dense, fine micro-channels within the matrix. Beyond 600 °C, the PPFs were completely volatilized, with no macroscopic fiber residues observable. The internal pores had significantly coarsened, and the matrix edges exhibited characteristic charred carbonization features. At 800 °C, the cross-section had fully carbonized to a deep black color, and the matrix presented a distinct loose granular structure, signifying the severe dehydration and decomposition of C-S-H gels and the consequent loss of binding capacity.

### 3.2. Mass Loss After High-Temperature Treatment

**Figure 8:**
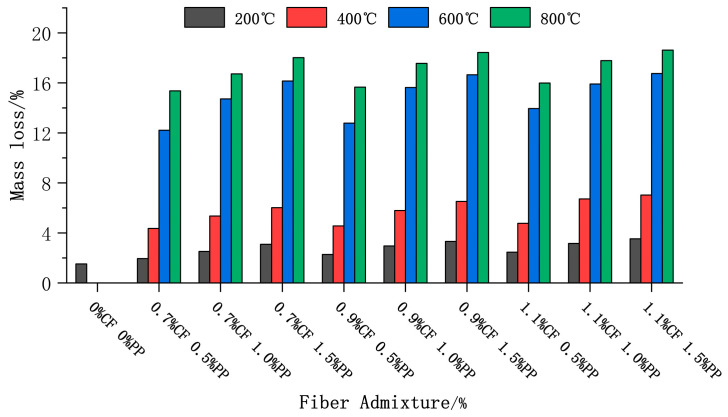
Mass loss of concrete after high-temperature treatment.

Figure 8 illustrates the mass loss rates of specimens with different mix proportions following high-temperature exposure. To minimize measurement errors induced by the size effect, prismatic specimens (100 mm × 100 mm × 400 mm) were selected for this evaluation. The results demonstrate that the mass loss rate followed a non-linear increasing trend with rising temperature, which can be categorized into three distinct stages:

Evaporation Stage (20–200°C): In this stage, mass loss was relatively minor, with a maximum reduction of approximately 3%. This is primarily attributed to the evaporation of free and capillary water within the matrix. Although physical melting of the PPFs occurred in this interval, extensive thermal pyrolysis and volatilization had not yet been initiated; thus, their contribution to the total mass loss was negligible.

Intense Pyrolysis Stage (400–600 °C): Within the 200–400 °C range, a pronounced increase in mass loss was observed; notably, the group containing 1.1% CF + 1.5% PP exhibited a loss of 7%. Beyond the continued escape of chemically bound water, this loss primarily involved the initial dehydration of C-S-H gel and the oxidative pyrolysis and volatilization of the PPFs [43]. In the 400–600 °C interval, the mass loss rate escalated steeply, peaking at 16.75% for the same group at 600 °C. The critical mechanism governing this phase is the severe dehydroxylation and decomposition of calcium hydroxide (CH) crystals, accompanied by extensive dehydration of the C-S-H gel and the complete depletion of residual free water [44]. The control group exhibited severe explosive spalling at temperatures exceeding 400 °C. Conversely, this phenomenon was effectively suppressed in the ECC group, facilitated by PPF melting, which created channels for vapor pressure release. The observed mass loss was thus dominated by water evaporation and chemical decomposition. This provides evidence of the excellent fire resistance of ECCs.

During this final stage, the rate of mass loss decelerated, with a further increment of approximately 2% observed. This loss mainly originated from the decomposition of residual C-S-H and the decarbonation of calcium carbonate (CaCO_3_).

Furthermore, a comparative analysis reveals that at a constant CF content, groups with higher PPF contents exhibited higher mass loss rates. The underlying mechanism behind this is twofold: apart from the direct mass loss caused by the pyrolysis of the PPFs themselves, the volatilization of a large amount of fibers left behind a vast network of interconnected pore channels. These channels intensified the inward heat transfer and increased the specific surface area of the matrix that was exposed to heat, thereby accelerating the thermal decomposition kinetics of the internal cementitious products.

### 3.3. Mechanical Properties

#### 3.3.1. Compressive Strength

**Figure 9:**
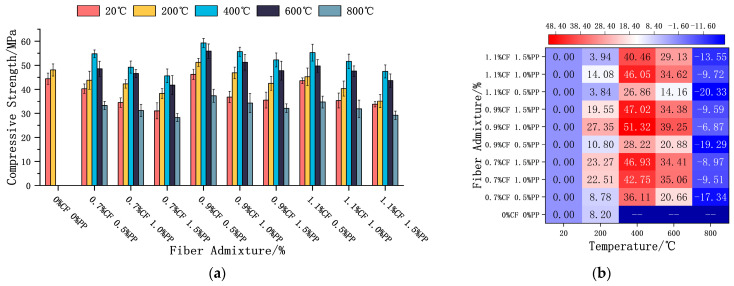
ECC compressive strength and strength loss after high-temperature treatment. (**a**) Effects of fiber admixture and temperature on the compressive strength of concrete. (**b**) Heatmap of the rate of compressive strength variation at different temperatures.

At ambient temperatures, the compressive strength of the ECC exhibited a monotonic declining trend with increasing PPF dosage. In contrast, increasing the CF dosage resulted in a non-monotonic “rise-and-fall” pattern, peaking at a dosage of 0.9% (Figure 9a). This divergence is primarily attributed to the distinct physical–mechanical properties of the two fibers. PPFs, characterized by a lower elastic modulus and a larger diameter, tend to introduce a weak Interfacial Transition Zone (ITZ) within the matrix. Under loading, these fibers undergo significant tensile deformation, failing to provide effective support. Conversely, CFs, possessing a micron-scale diameter and a high modulus, are dispersed more uniformly, significantly enhancing matrix stiffness through the micro-filler effect and bridging actions. However, when the CF content is too high (reaching 1.1%), fiber agglomeration intensifies. The resulting localized stress concentration points counteract the benefits of reinforcement, leading to a regression in compressive strength.

Despite the differences in initial strength among the different groups, their property trends with rising temperature exhibited high consistency (Figure 9b). In the 20 °C to 400 °C range, the compressive strength of all specimens improved significantly, with increments ranging from 26.86% (1.1% CF + 0.5% PPF) to 51.32% (0.9% CF + 1.0% PPF). However, as the temperature further increased to the 600–800 °C range, a steep decline in strength was observed. After exposure to a temperature of 800 °C, the residual strength of all groups fell below their ambient baselines, maintaining a level of approximately 30 MPa. Notably, the deterioration was most pronounced in the 1.1% CF + 0.5% PPF group, where the residual strength (34.71 MPa) exhibited a loss of approximately 20.33% compared to the ambient state.

The dual influence of temperature on compressive strength is essentially a result of the competition between the “high-temperature strengthening mechanism” and the “thermally induced damage mechanism.” During the 20–400 °C stage, unreacted active particles (such as fly ash and silica fume), abundant in the ECC system, underwent a significant “internal autoclaving effect” under the combined action of high temperature and internal pore water pressure. This process promoted secondary hydration reactions, generating high-density C-S-H gels that effectively filled micro-cracks and capillary pores, thereby enhancing the density and load-bearing capacity of the matrix. However, when the temperature exceeded 600 °C, as the potential for secondary hydration was exhausted, the dehydration/decomposition of C-S-H gels and the thermal incompatibility at the aggregate interface began to dominate. Simultaneously, the melting and volatilization of PPFs left behind numerous in situ interconnected channels. While beneficial for spalling resistance, these defects weakened the fiber bridging effect and facilitated rapid crack propagation, ultimately leading to a significant reduction in compressive strength.

#### 3.3.2. Splitting Tensile Strength

**Figure 10:**
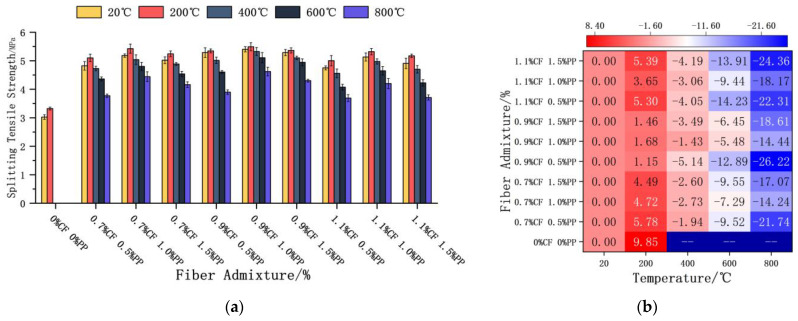
ECC splitting tensile strength and strength loss after high-temperature treatment. (**a**) Effects of fiber admixture and temperature on the splitting tensile strength of concrete. (**b**) Heatmap of the rate of splitting tensile strength variation at different temperatures.

Under varying temperature conditions, the PPFs and CFs demonstrated significant hybrid synergistic reinforcement effects (Figure 10). The contribution of both fiber types to tensile performance followed a non-monotonic “rise-and-fall” pattern, with the optimal synergistic ratio stabilizing at 1.0% PPF + 0.9% CF. Compared to the plain concrete control group at ambient temperature, this optimal combination achieved strength enhancements of 78.6%, 81.6%, 76.1%, 68.8%, and 52.8% at the respective test temperatures (Figure 10a). This result confirms that the hybrid fiber system effectively mitigates crack propagation through bridging action across the full temperature range, significantly improving the tensile bearing capacity of the matrix. However, a distinct dose threshold exists for fiber reinforcement. When the dose is too high, fiber agglomeration tends to occur within the matrix. This agglomeration hinders the effective encapsulation of fibers by hydration products, thereby compromising the interfacial bonding properties. Under tensile loading, these agglomerated zones often act as stress concentration points, leading to premature fiber pull-out and a consequent regression in mechanical performance.

The ECC’s splitting tensile strength also exhibited a “rise-and-fall” evolutionary trend as the temperature increased, but notably, the peak temperature was 200 °C. As the temperature rose from 400 °C to 800 °C, the strength exhibited a significant, approximately linear decline. Taking the optimal group (1.0% PPF + 0.9% CF) as an example: compared to its 20 °C baseline, the strength increased by 1.68% at 200 °C, but decreased by 1.43%, 5.58%, and 14.4% at 400 °C, 600 °C, and 800 °C, respectively (Figure 10b). Following exposure to the extreme temperature of 800 °C, the 0.5% PPF + 0.9% CF group suffered the most severe loss (26.22%), indicating that a low dosage of PPFs is insufficient to maintain matrix integrity under such conditions.

In the 20–200 °C interval, unhydrated cementitious particles within the matrix underwent secondary hydration reactions triggered by the high-temperature steam environment. This process densified the fiber–matrix Interfacial Transition Zone. At this stage, only a small amount of near-surface PPFs had melted; the strength gains from secondary hydration outweighed the negative impact of incipient melting, resulting in a macroscopic increase in splitting tensile strength. However, beyond 200 °C, the strengthening mechanism failed, and deterioration mechanisms began to dominate. The PPFs completely melted rapidly and were removed from the load-bearing system, causing a total loss of bridging action. Simultaneously, thermally induced stress cracks propagated and became interconnected. The superposition effect of these factors directly led to the continuous decline in splitting tensile strength.

#### 3.3.3. Flexural Strength

**Figure 11:**
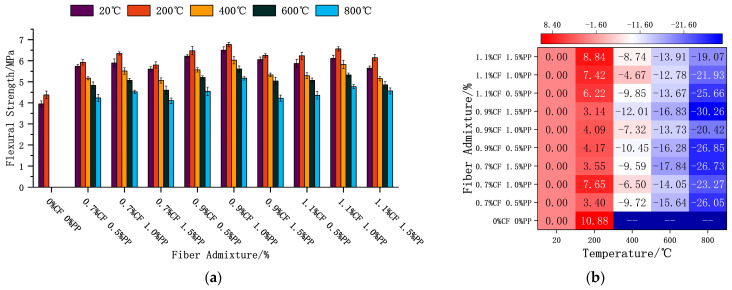
ECC flexural strength and strength loss after high-temperature treatment. (**a**) Effects of fiber admixture and temperature on the flexural strength of concrete. (**b**) Heatmap of the rate of flexural strength variation at different temperatures.

Figure 11 summarizes the evolution of flexural strength for specimens with varying mix proportions after high-temperature exposure. At all test temperatures, the influence of both fiber types on flexural strength followed a non-monotonic “rise-and-fall” trend, with 1.0% PPF + 0.9% CF identified as the optimal synergistic ratio. At ambient temperature (20 °C), this optimal group and the lowest-performing group (1.5% PPF + 0.7% CF) exhibited strengths 54.5% and 41.8% higher, respectively, than that of the plain concrete control (Figure 11a). This fully confirms the significant advantages of the hybrid fiber system in enhancing flexural bearing capacity. However, a clear dosage threshold exists for the fiber reinforcement effect. Excessive fiber incorporation not only reduces matrix compactness but also hinders the effective encapsulation and wetting of fibers by hydration products due to fiber agglomeration. This weakening of the interfacial bond prevents the fibers from fully exerting their bridging and toughening mechanisms under flexural–tensile loading, resulting in macroscopic strength regression.

The temperature dependency of flexural strength also exhibited a “rise-and-fall” pattern, with a peak observed at 200 °C. At this temperature, the strength of the optimal group (1.0% PPF + 0.9% CF) and the lowest group (1.5% PPF + 0.7% CF) had increased by 4.09% and 3.55%, respectively, relative to their own 20 °C baselines. At temperatures between 400 °C and 800 °C, the flexural strength gradually decayed. Remarkably, however, even after exposure to 800 °C, the residual flexural strength of all fiber-reinforced groups remained higher than that of the plain concrete control at 20 °C. Specifically, the lowest strength value recorded at 800 °C (for 1.5% PPF + 0.7% CF) was still 3.87% higher than the ambient control. This observation serves as potent evidence that even after the complete melting and failure of the PPFs, the heat-resistant CFs continue to provide critical skeletal support and reinforcement within the matrix. The maximum (30.26%) and minimum (19.07%) loss rates at 800 °C compared to each group’s 20 °C baseline were observed for the 1.5% PPF + 0.9% CF and 1.5% PPF + 1.1% CF groups, respectively (Figure 11b).

This temperature dependency essentially reflects the mechanisms of microstructural evolution. In the 20–200 °C stage, high-temperature-activated secondary hydration reactions improved the matrix microstructure, promoting tighter encapsulation between hydration products and fiber surfaces. The resulting densification of the concrete interior enhanced the overall flexural performance. However, as the temperature rose further, the PPFs gradually melted, leaving behind in situ voids, which signified the failure of the physical bridging action. Under loading, the coalescence of these melting-induced voids with thermally induced micro-cracks rapidly evolved into propagating cracks, ultimately leading to a significant reduction in flexural strength.

#### 3.3.4. Flexural Toughness

**Figure 12:**
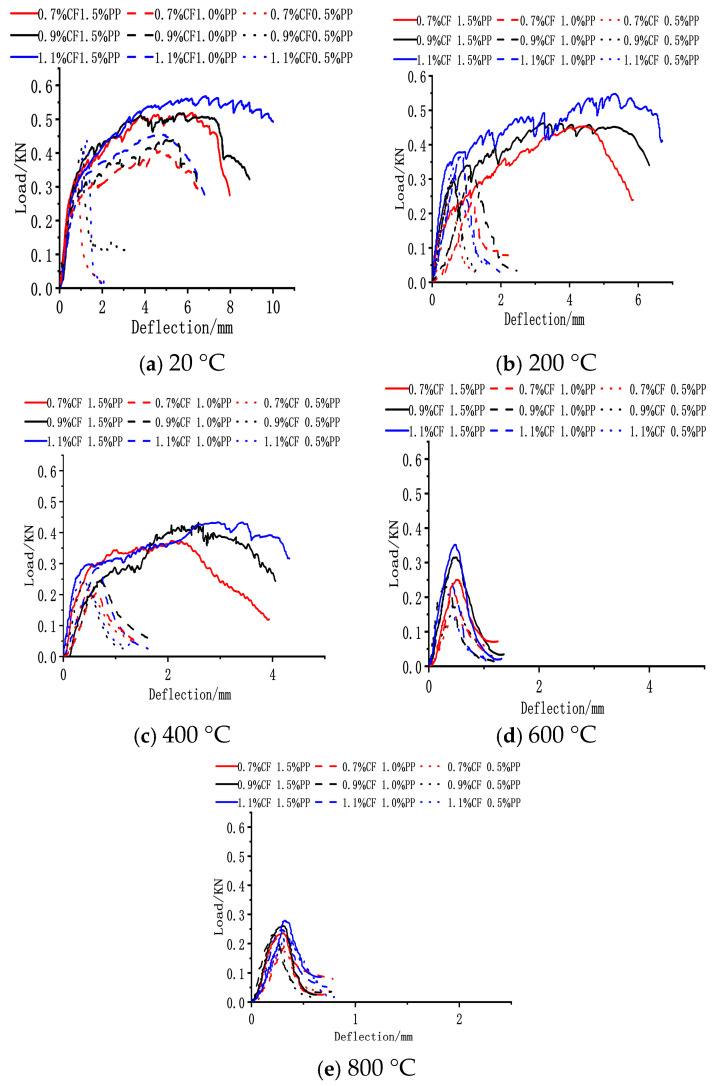
Load–deflection curves of CF-PP-reinforced specimens at different temperatures.

The experimental temperature and PPF volume fraction exerted a significant influence on the flexural toughness of the ECC. As illustrated in Figure 12, the flexural toughness of the ECC improved notably with increasing PPF contents, whereas the incorporation of CFs primarily enhanced the matrix’s first-crack strength and ultimate strength. At a 0.5% PPF dosage, the ECC plates exhibited brittle failure characteristics across all test temperatures. When the dosage was increased to 1.0%, the specimens displayed distinct strain-hardening behavior at 20 °C but transitioned to brittle failure upon heating to 200 °C; this indicates that the critical PPF dosage required to maintain strain-hardening potential under high-temperature conditions lies between 0.5% and 1.0%. Notably, at 1.5% PPFs, the ECC plates maintained excellent ductility within the 20 °C to 400 °C range, shifting to brittle failure only when the temperature reached 600 °C.

**Figure 13:**
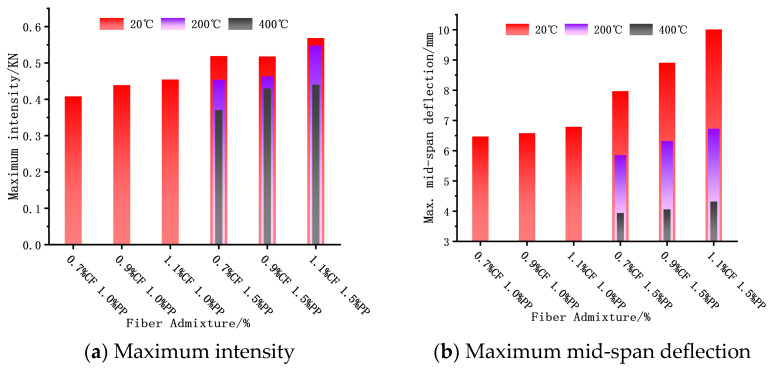
Maximum intensity and maximum mid-span deflection.

At a constant PPF dosage, the inclusion of carbon fibers significantly elevated the ultimate strength of the concrete (Figure 13). At 20 °C with 1.5% PPFs, increasing the CF content from 0.7% to 1.1% resulted in a 9.6% increase in ultimate strength. This enhancement is attributed to the effective combination of high-elastic-modulus CFs with the matrix, which improved the overall composite stiffness and synergistically boosted both the first-crack and ultimate strengths. However, for the specimens with 1.5% PPFs, the test temperature exerted a significant degrading effect on flexural toughness. With rising temperature, both the ultimate strength and maximum mid-span deflection of the ECC plates showed a clear declining trend. Taking the optimal toughness group (1.5% PPF + 1.1% CF) as an example, after exposure to 200 °C and 400 °C, its ultimate strength decreased by 3.6% and 22.6%, and the maximum mid-span deflection reduced by 32.8% and 56.8%, respectively, compared to the ambient baseline (20 °C).

The reduction in flexural toughness under high-temperature environments is driven by the combined action of two mechanisms. First, the melting of PPFs at high temperatures increases the porosity within the matrix, reducing its compactness and weakening the fiber bridging effect. Second, and more critically, high-temperature-activated secondary hydration reactions significantly densify the fiber–matrix interface, enhancing the chemical bonding force. This excessive bonding causes a shift in the fiber failure mode under external loading from the energy-dissipating “slip and pull-out” mode to the brittle “fiber rupture” mode, thereby drastically reducing the ductility and flexural toughness of the material.

### 3.4. Self-Sensing Performance

#### 3.4.1. ECC Resistance After High-Temperature Treatment

**Figure 14:**
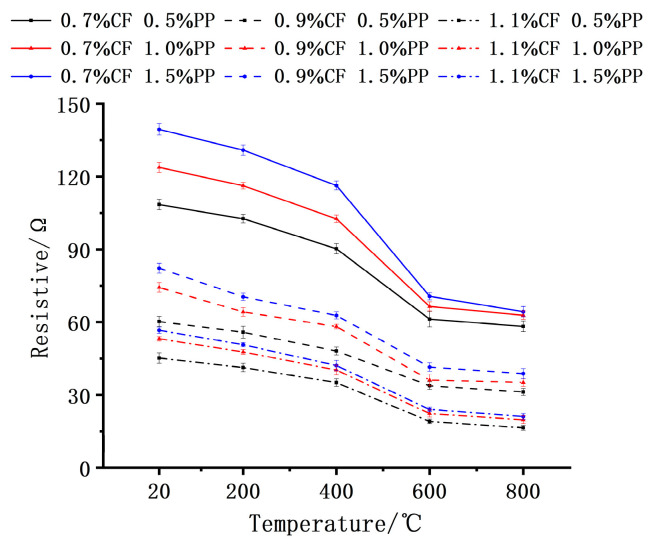
Variations in electrical resistivity with temperature for specimens with different CF and PP contents.

The influence of high temperature on the electrical resistance of the concrete is illustrated in Figure 14. Generally, the electrical resistance exhibited a declining trend with increasing test temperature. In the range of 20 °C to 400 °C, the rate of resistance reduction was relatively moderate. Comparing the values at 400 °C to the 20 °C baseline, the maximum and minimum reduction rates were observed for the 1.1% CF + 1.5% PPF and 0.7% CF + 1.5% PPF groups, with decreases of 29.4% and 16.6%, respectively. However, when the temperature rose to 600 °C, the resistance values of all mix proportions experienced an abrupt decline. The 1.1% CF + 1.5% PPF group exhibited the most significant drop, reaching 59.7%. Beyond 800 °C, the resistance values tended to stabilize, with the variation amplitude controlled within 10% compared to the 600 °C level.

The evolution of electrical resistance with respect to temperature and fiber content is primarily jointly governed by fiber network reconstruction and thermal activation effects. Specifically, regarding the influence of carbon fiber (CF) content, a high CF content leads to a more robust conductive network within the matrix, where elevated temperatures significantly lower the potential barrier for electron tunneling between fibers and activate a large number of potential conductive pathways. Therefore, in the high-CF group, a greater magnitude of resistance decrease is observed. Regarding the influence of PPF content, PPFs act as insulators at ambient temperature that impede the overlapping of carbon fibers; however, above a certain temperature, the PPFs melt and evaporate. The resulting in situ voids lead to the elimination of these physical barriers, prompting previously unconnected CFs to contact and overlap into conductive channels. Consequently, a higher PPF content results in a larger volume of insulation being removed after high-temperature melting and a more significant recovery effect of the conductive network, which explains why the group with high PPF content exhibits the largest sudden drop in resistance after 600 °C. Upon reaching 800 °C, although the conductive network tends to stabilize, thermally induced matrix cracks disrupt partial conductive paths, limiting further the decline in resistance and causing the values to plateau.

#### 3.4.2. Temperature Sensitivity

**Figure 15:**
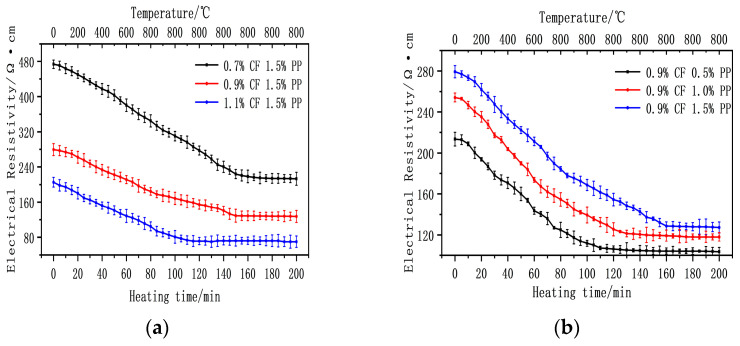
Effect of fiber content on ECC temperature sensitivity. (**a**) Effect of different CF contents on temperature-sensitive properties. (**b**) Effect of different PPF contents on temperature-sensitive properties.

Figure 15a illustrates the significant influence of CF dosage on the temperature sensitivity of the composites. During the heating phase (0–80 min), the electrical resistivity of all specimens exhibited a monotonic decreasing trend with increasing temperature. At CF contents of 0.7%, 0.9% and 1.1%, the Fractional Change in Resistivity (FCR) was −27.1%, −34.0%, and −49.1%, respectively, indicating that higher CF contents significantly enhance the thermal sensitivity of the material. This difference in sensitivity is primarily attributed to the ‘thermally activated electron tunneling’ mechanism. In the system with a high CF content (1.1%), a denser percolation network is established within the matrix, containing numerous fiber nodes in a ‘critical contact’ state. When external thermal energy provides the activation energy required to overcome the interfacial potential barriers, electrons can efficiently tunnel between these critical nodes. In contrast, specimens with a low CF content possess fewer conductive pathways; consequently, the contribution of thermal excitation to the overall conductivity is limited, resulting in lower sensitivity.

Figure 15b reveals the influence of different PPF contents on temperature sensitivity. During the heating phase, specimens with a lower PPF content exhibited a higher rate of decline in resistivity, with the fractional changes reaching 41.5%, 39.2%, and 34.0%, respectively. Although the impact of PPF content on the magnitude of the final resistivity change was limited (with a maximum difference of only 7.5%), a high PPF dosage significantly prolonged the time required to reach equilibrium during the isothermal phase, exhibiting a distinct stabilization lag in thermal sensitivity.

**Figure 16:**
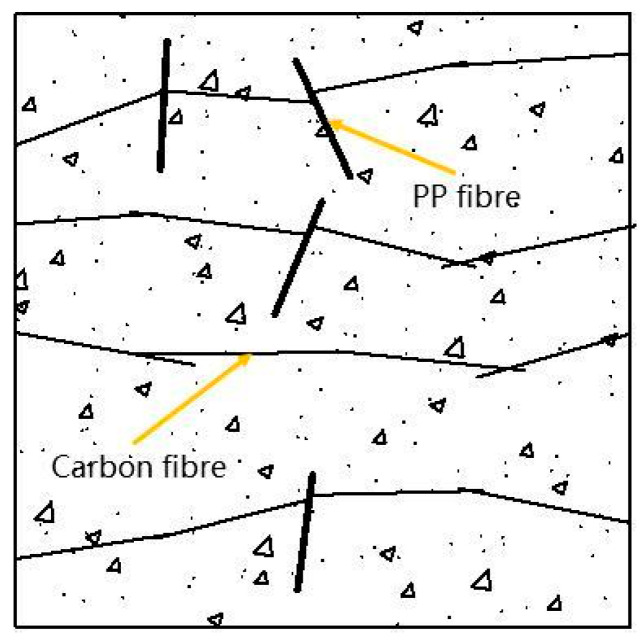
State of fiber dispersion within concrete.

Fundamentally, this stabilization lag in thermal sensitivity is determined by PP fibers acting as ‘thermal physical switches,’ the mechanism of which is illustrated in Figure 13. At ambient temperature, the dispersed insulating PP fibers physically impede the continuous distribution of carbon fibers, thereby severing potential conductive pathways (Figure 16). As the temperature rises and exceeds the melting point of the PPFs, these physical barriers are gradually eliminated through phase change, prompting the previously separated carbon fibers to reconnect under the influence of thermal expansion. However, this reconstruction process is not instantaneous. A higher PPF dosage implies the presence of a larger volume of ‘insulating barriers’ within the matrix that must be removed via melting and diffusion. Consequently, this manifests in the thermal sensitivity performance as a significant lag in the stabilization time of the conductive network for specimens with a high PPF content.

#### 3.4.3. Pressure Sensitivity

**Figure 17:**
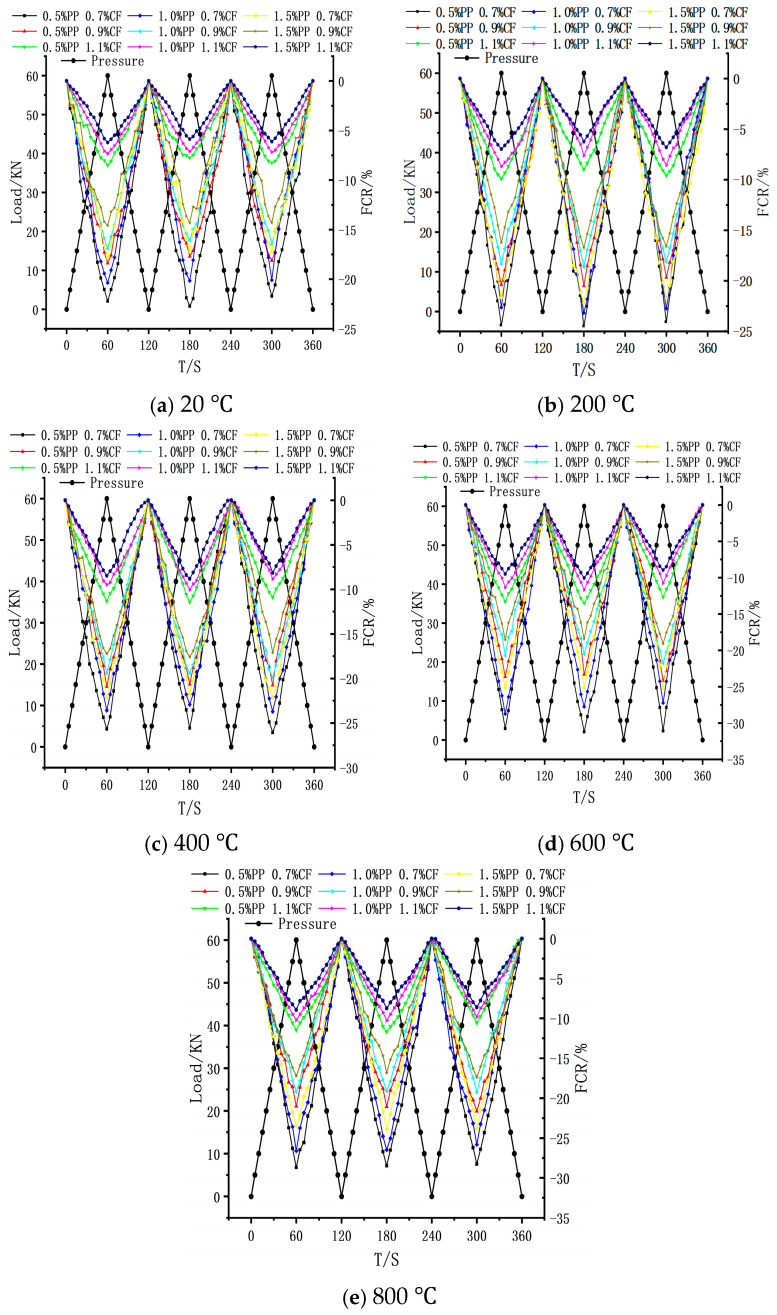
Pressure sensitivity of ECC after exposure to different test temperatures.

Figure 17 illustrates the pressure sensitivity characteristics of the concrete under various test temperatures. Overall, the PPF dosage exhibits an inverse correlation with pressure sensitivity. Across all temperature gradients, the pressure sensitivity of specimens with different CF dosages is ranked as follows: 0.7% > 0.9% > 1.1%. Consequently, the 0.7% CF + 0.5% PPF combination exhibited the optimal sensing performance. The influence of test temperature on pressure sensitivity followed a non-monotonic “rise-and-fall” trend: In the 20 °C to 600 °C range, pressure sensitivity was significantly enhanced with increasing temperature. At 600 °C, the optimal group (0.7% CF + 0.5% PPF) achieved a maximum Fractional Change in Resistivity (FCR) of 31.1%, representing a 37% increase compared to the value at 20 °C. However, in the 600 °C to 800 °C stage, the maximum FCR gradually decayed, and by 800 °C, the pressure sensitivity of all groups fell slightly below ambient levels.

**Figure 18:**
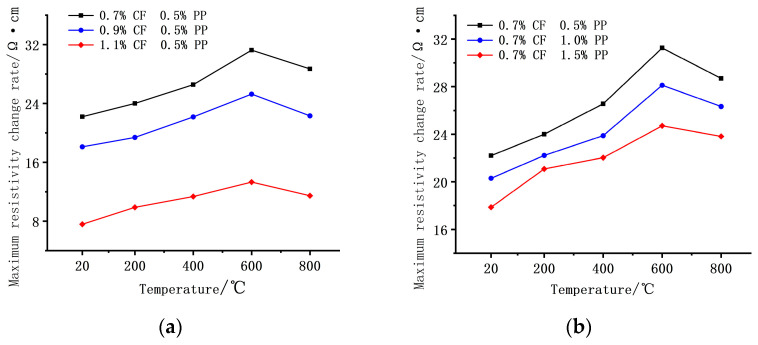
Effect of fiber dosage on pressure sensitivity. (**a**) Pressure-sensitive properties at different CF contents. (**b**) Pressure-sensitive properties at different PPF contents.

Figure 18a reveals the significant impact of CF dosage on pressure sensitivity. Under a constant 0.5% PPF dosage and across all temperature gradients, the maximum FCR of the 0.7% CF group was substantially higher than that of the 1.1% CF baseline group, with enhancement amplitudes ranging from 134.0% to 192.4%. This mechanism can be explained by Conductive Percolation Theory: the change in pressure sensitivity originates from the increased contact between internal CFs and the reconstructed conductive pathways induced by volume shrinkage under compression. When the CF dosage is 0.7% (close to the percolation threshold), the matrix contains a vast number of “potential” conductive pathways in a critical contact state; thus, even minute deformations can trigger these paths to connect, resulting in a drastic change in resistance. Conversely, as the CF dosage increases to 0.9% and 1.1%, the CF network tends toward saturation, where initial conductive pathways are already well established, leading to a diminishing marginal gain in network connectivity under external compression and a consequent reduction in pressure sensitivity.

Increasing the PPF dosage reduced the maximum FCR. Taking the 0.5% PPF group as an example, its maximum FCR increased by 24.3%, 13.9%, 20.1%, 26.5%, and 20.4% at the respective temperatures compared to the 1.5% PPF group (Figure 18b). At ambient temperature, the large-diameter, insulating PPFs exerted a “screening effect,” creating physical barriers that impeded CF overlap and inhibited the formation of conductive pathways under compression. Tests indicate that even after the PPFs melted at beyond 600 °C, the pressure sensitivity of the high-PPF group remained inferior to that of the low-dosage group. This is primarily attributed to two factors: First, the voids left by the melting of a large volume of PPFs tended to expand and coalesce into micro-cracks under load, causing the rupture or dislocation of adjacent carbon fibers and destabilizing the conductive network. Second, the increased brittleness of the matrix due to high-temperature exposure limited the material’s ultimate compressive strain, thereby reducing the magnitude of the deformation-based resistance response.

#### 3.4.4. Flexural Sensitivity

**Figure 19:**
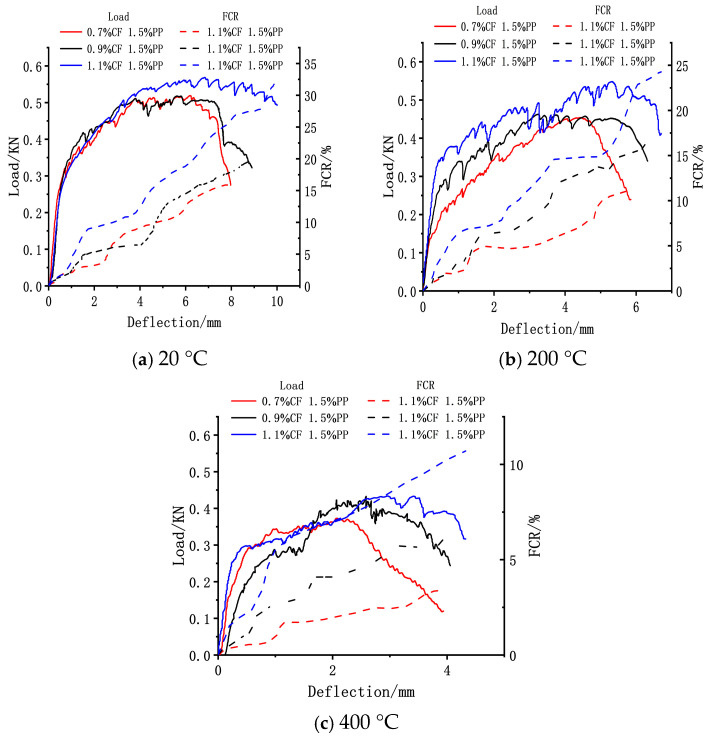
Flexural sensitivity of ECCs with different fiber admixtures.

The flexural sensing performance of ECC plates containing 1.5% PPF was evaluated under various temperature gradients, with the data summarized and presented in Figure 19. At all test temperatures, the concrete exhibited distinct flexural sensitivity. Notably, the flexural sensitivity gradually improved with increasing CF dosage. At 20 °C, the specimen with 1.1% CFs demonstrated a significant performance enhancement, with its maximum Fractional Change in Resistivity (FCR) increasing by 18.7% compared to the sample containing 0.7% CFs. The flexural self-sensing mechanism of the concrete is primarily governed by the progressive disruption effect of conductive pathways on the tensile side. A higher CF dosage implies the formation of a denser initial conductive network within the matrix; during flexural loading, as the mid-span deflection increases, crack propagation causes the debonding or rupture of previously overlapping carbon fibers. This physical truncation of conductive pathways leads to a sharp surge in resistivity, thereby endowing the material with superior flexural sensitivity at higher CF dosages.

However, rising test temperatures exerted a significant deteriorating effect on flexural sensitivity. Using the 20 °C data as the baseline, after exposure to 200 °C and 400 °C, the maximum FCR of specimens with three different CF dosages (0.7%, 0.9%, and 1.1%) exhibited varying degrees of attenuation. Specifically, the reductions were 30.8%, 16.7%, and 24.3% at 200 °C and escalated to 78.8%, 67.7%, and 66.6% at 400 °C, respectively. This degradation is attributed to the high-temperature-induced melting of PPFs and matrix embrittlement, which significantly reduced the flexural toughness and ultimate deformation capacity of the ECC. The reduced mid-span deflection restricted the full propagation of cracks and the extent of conductive network destruction, consequently leading to a diminished resistivity response.

## 4. Micro Analysis

### 4.1. Scanning Electron Microscope

**Figure 20:**
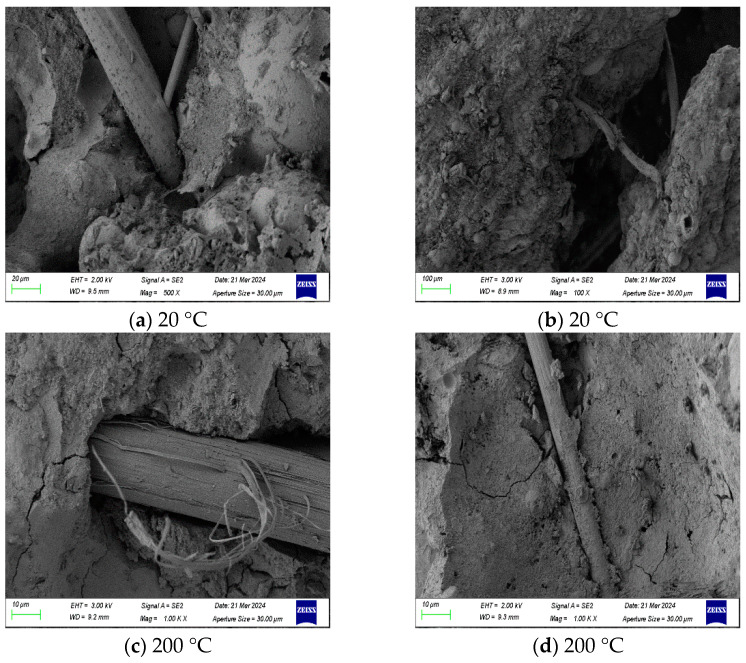

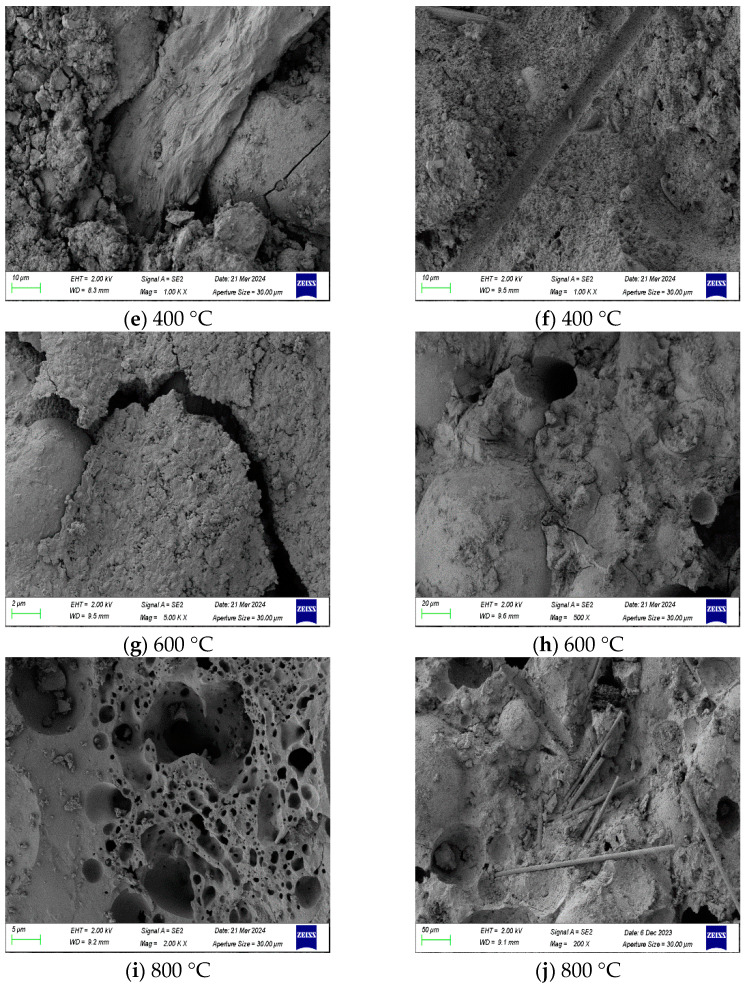
Scanning Electron Microscopy (SEM) images of ECC microstructures after exposure to different temperatures.

Figure 20 illustrates the microstructural morphology of the ECC after exposure to different test temperatures. As clearly evident from Figure 20a, the presence of polypropylene fibers (PPFs) exerted a significant influence on the spatial distribution of carbon fibers (CFs). The bulky PPFs physically impeded the continuity of the CF network, which explains the inferior electrical and self-sensing performance observed in the high-PPF dosage groups. Figure 20b demonstrates the bridging action of fibers across cracks. Under external loading, these fibers effectively transferred internal stresses and inhibited crack propagation, thereby enhancing the flexural toughness of the concrete. Meanwhile, unhydrated fly ash particles were visible in the micrographs at 20 °C and 200 °C; the secondary hydration of these particles contributes to increasing the internal compactness of the matrix, consequently improving the mechanical properties of the concrete.

When the temperature rose to 200 °C, the PPFs exhibited incipient melting and deformation (Figure 20c), which to a certain extent weakened their bridging capacity. However, SEM images revealed that the high temperature promoted the enrichment and growth of secondary hydration products on the fiber surface (Figure 20d). This improved the fiber–matrix interfacial bonding, explaining why certain mechanical properties were enhanced in this temperature range. Upon reaching 400 °C (Figure 20e), extensive PPF melting occurred, leaving voids within the concrete. The few remaining PPFs exhibited significant thermal shrinkage (Figure 20f) and lost their mechanical functionality, failing to effectively transfer stress. Upon melting, PPFs effectively form an interconnected pore network, acting as a pressure relief channel, thereby preventing explosive spalling of the high-strength ECC matrix [45]. Consequently, at this temperature, the ECC plates lost their flexural toughness and transitioned to brittle failure.

At 600 °C, the microstructure underwent severe degradation, initiating the proliferation of micro-cracks within the matrix (Figure 20g). At this stage, the voids left by fiber melting acted as stress concentrators under an external load, inducing the coalescence of voids into macroscopic cracks and leading to a drastic decline in mechanical performance (Figure 20h). At the extreme temperature of 800 °C, the microstructure deteriorated further (Figure 20i). The decomposition of hydration products resulted in a loose, honeycomb-like porous structure, causing an abrupt drop in density and strength. However, the CFs demonstrated excellent thermal stability, maintaining structural integrity without significant melting even at 800 °C (Figure 20j). This thermal resilience provided crucial support, allowing the ECC to retain a certain degree of residual strength post-exposure.

### 4.2. XRD Analysis

**Figure 21:**
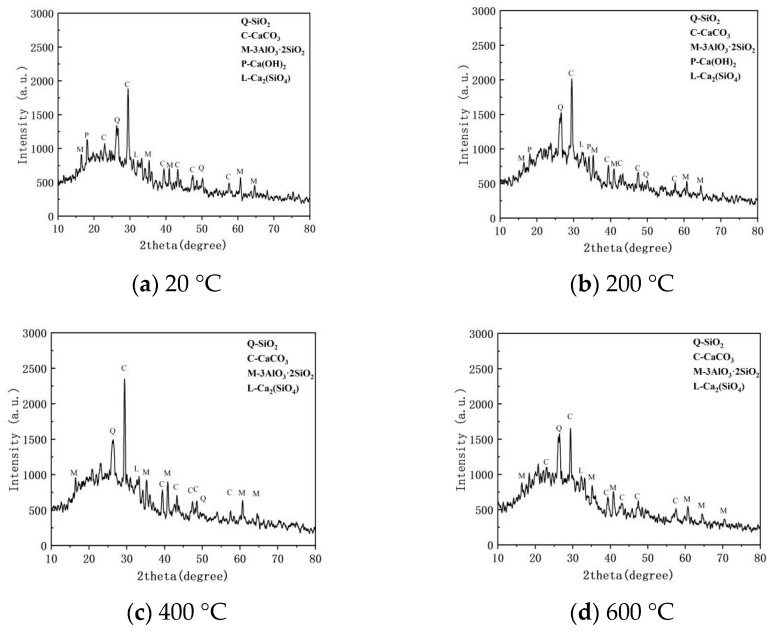
XRD patterns of ECC specimens after exposure to different temperatures.

Figure 21 illustrates the X-ray Diffraction (XRD) patterns of the ECC matrix after exposure to various high temperatures, revealing the thermal evolution of the internal crystalline phases. The patterns across all temperature gradients displayed strong diffraction peaks corresponding to Quartz (Q-SiO_2_) and Mullite (M-3Al_2_O_3_·2SiO_2_). The Quartz primarily originated from the glass sand aggregates and unreacted siliceous raw materials, while Mullite is a typical high-temperature-stable phase derived from fly ash. Notably, even at 800 °C, the strength and position of the Q and M diffraction peaks remained highly stable. This indicates that the glass sand and fly ash possess excellent volumetric stability and chemical inertness in high-temperature environments, providing a rigid thermal support skeleton for the ECC. At 200 °C (Figure 21a), distinct characteristic peaks of Portlandite (Ca(OH)_2_) and Calcite (C-CaCO_3_) appeared in the spectrum, indicating that the structure of hydration products within the matrix remained intact at this stage. However, as the temperature rose to 400 °C and above (Figure 21b–d), the characteristic diffraction peaks of Portlandite (2θ ≈ 18°and34°) vanished completely. This is attributed to the intense dehydroxylation reaction of calcium hydroxide within the 400–500 °C interval [46,47,48]. This reaction induced micro-cracking and increased porosity within the matrix, which aligns with the decline in mechanical properties discussed earlier.

Although the theoretical thermal decomposition temperature of calcium carbonate (Calcite) is around 700–800 °C, the diffraction peaks of Calcite in the 800 °C spectrum (Figure 18b) remained sharp and high in strength. This phenomenon is primarily attributed to the post-heating “Recarbonation Effect.” While a portion of CaCO_3_ decomposed into Calcium Oxide (CaO) at 800 °C, this CaO possesses extremely high chemical reactivity. During the cooling and storage process of the specimens, it readily absorbed CO_2_ from the air and rapidly reverted to secondary CaCO_3_. Furthermore, the emergence of Larnite (L-Ca_2_SiO_4_) peaks in the spectrum indicates that the high temperatures induced the dehydration of amorphous C-S-H gel and its transformation into crystalline β-C_2_S. While this “ceramization” phase transformation contributed to an increase in matrix brittleness, it also played a crucial role in retaining the material’s degree of residual strength after exposure to extreme heat.

## 5. Conclusions

To address the limitations of traditional concrete, specifically its susceptibility to high-temperature spalling, and the lack of in situ monitoring methods, in this study, a novel self-sensing, high-temperature-resistant ECC was developed by fully replacing silica sand with eco-friendly glass sand and incorporating a hybrid PPF/CF system. Based on a systematic investigation of mechanical evolution, multi-functional self-sensing characteristics, and micro-damage mechanisms after thermal treatment from 20 °C to 800 °C, the following conclusions are drawn:

(1) The incorporation of hybrid fibers effectively mitigated explosive spalling up to 800 °C via the “pore pressure relief” mechanism, whereas plain concrete disintegrated at 400 °C. Mechanical properties followed a non-monotonic evolutionary trend with increasing temperature. The optimal synergy for compressive strength was achieved with a mixture of 0.9% CF and 1.0% PPF, which yielded a peak increase of approximately 51% at 400 °C. Conversely, the splitting tensile and flexural strengths reached their maximum at 200 °C, where the combination of 1.0% PPF and 0.9% CF proved to be the most effective formulation. Even at 800 °C, the carbon fiber skeleton maintained residual strength superiority over plain concrete.
(2) PPFs dominated energy dissipation and toughening, while CFs primarily enhanced first-crack strength and ultimate load. A clear ductile-to-brittle transition occurred above 400 °C due to fiber melting and matrix embrittlement. Notably, specimens containing 1.5% PPF retained pseudo-strain-hardening characteristics within the 20–400 °C range, identifying this specific dosage as critical for ductility retention in moderate fire scenarios. Uniaxial tensile stress–strain curves were not obtained due to the brittleness of the specimens following high-temperature exposure. Future experiments will utilize Digital Image Correlation (DIC) techniques for in-depth validation.
(3) The composite demonstrated multimodal sensing capabilities, though the optimal fiber dosages varied significantly by sensing mode. A high fiber content consisting of 1.1% CF and 1.5% PPF provided the superior thermosensitivity (49.1% resistivity reduction) required for early fire warnings. In contrast, a lower dosage of 0.7% CF combined with 0.5% PPF achieved the highest pressure sensitivity at 600 °C. However, flexural sensitivity suffered significant attenuation at elevated temperatures due to limited crack propagation capacity.
(4) Microstructural analyses confirmed that the melting of PPFs created interconnected channels for vapor release, serving as the key mechanism for spalling suppression. The severe dehydroxylation of calcium hydroxide (CH) in the 400–500 °C interval was identified as the chemical trigger for mechanical degradation. Furthermore, carbon fibers remained structurally stable at 800 °C, thereby preserving the conductive network essential for post-fire damage assessment.

## Figures and Tables

**Table 1:** Physical and chemical properties of cement.

Properties		Standard Value	Actual Value
Physical properties	Specific surface area (m^2^/kg)	≥300	329
	Initial set (min)	≥45	192
	Final set (min)	≤600	240
Compressive strength	3 days (MPa)	≥17.0	27.7
	28 days (MPa)	≥42.5	48.7
Flexural strength	3 days (MPa)	≥3.5	5.2
	28 days (MPa)	≥6.5	7.6
Chemical properties	Loss on ignition (%)	≤5.0	3.64
	MgO (%)	≤5.0	1.01
	CaO (%)	≥66.0	66.54
	SiO_2_ (%)	≥20.0	21.03
	Al_2_O_3_ (%)	≥4.0	4.36
	Fe_2_O_3_ (%)	≥2.0	2.32
	CaSO_4_·2H_2_O (%)	≥2.0	2.14
	SO_3_ (%)	≤3.5	2.16
	Cl^−^ (%)	≤0.06	0.021

**Table 2:** Physical and chemical properties of silica fume.

Properties		Standard Value	Actual Value
Physical and chemical properties	SiO_2_ (%)	≥90.0	90.41
	MgO (%)	-	0.71
	Al_2_O_3_(%)	-	1.04
	CaO (%)	-	0.27
	Fe_2_O_3_ (%)	-	0.32
	Loss on ignition (%)	≤2.0	1.37
	Cl^−^ (%)	≤2.0	0.125
	PH	4.0∼8.5	6.8
	Moisture content (%)	≤3.0	0.65
	Water demand ratio (%)	≤125	117

**Table 3:** Physical and chemical properties of fly ash.

Properties		Standard Value	Actual Value
Physical properties	Fineness (%)	≤12	10.6
Chemical properties	Water demand ratio (%)	≤95	93
	Loss on ignition (%)	≤5.0	0.77
	Moisture content (%)	≤1.0	0.11
	SiO_2_ (%)	≥40.0	57.33
	Al_2_O_3_ (%)	≥10.0	18.25
	Fe_2_O_3_ (%)	≥2.0	5.65
	CaO (%)	≥5.0	6.17
	SO_3_ (%)	≤3.0	0.10
	CaO_3_ (%)	≤1.0	0.68
	Strong activity index (%)	≥70	74

**Table 4:** Chemical composition of glass sand.

SiO_2_ (%)	KCl (%)	Na_2_O (%)	CaO (%)	MgO (%)	Fe_2_O_3_ (%)	Al_2_O_3_ (%)
72.81	0.72	13.35	8.74	1.15	0.18	2.62

**Table 5:** Polypropylene fiber performance indicators.

Diameter (μm)	Lengths (mm)	Density (g/mm^3^)	Tensile Strength (MPa)	Elongation at Break (%)	Melting Point (°C)
20	12	0.91	680	18	165

**Table 6:** Carbon fiber performance indicators.

Diameter (μm)	Lengths (mm)	Density (g/mm^3^)	Tensile Strength (MPa)	Elongation at Break (%)	Carbon Content	Modulus of Elasticity (GPa)
6	6	1.79	3950	1.45	95.9%	238

**Table 7:** Performance index of water-reducing agent.

Test Items	Standard Value	Actual Value
Water reduction rate (%)	≥25	30
Gas content (%)	≤6.0	3.0
Normal pressure water secretion ratio (%)	≤60	10
Na_2_SO_4_ (%)	≤5.0	0.6
Cl^−^ (%)	≤0.6	0.03
Total alkali content (%)	≤10	1.12
Shrinkage ratio (%)	≤110	102
pH	5.0 ± 1.0	5.2
Density g/cm^3^	1.06 ± 0.02	1.06
Solid content (%)	38 ± 1.9	38

**Table 8:** Mixing ratio design.

Batch Number	Cement	Fly Ash	Silica Fume	Glass Sand	Water	CFs	PPF
N0	480	576	144	432	360	-	-
N1	480	576	144	432	360	0.7%	0.5%
N2	480	576	144	432	360	0.7%	1.0%
N3	480	576	144	432	360	0.7%	1.5%
N4	480	576	144	432	360	0.9%	0.5%
N5	480	576	144	432	360	0.9%	1.0%
N6	480	576	144	432	360	0.9%	1.5%
N7	480	576	144	432	360	1.1%	0.5%
N8	480	576	144	432	360	1.1%	1.0%
N9	480	576	144	432	360	1.1%	1.5%

## Data Availability

The original contributions presented in this study are included in the article. Further inquiries can be directed to the corresponding author.

## Abbreviations

The following abbreviations are used in this manuscript:

ECC	Engineered cementitious composite
PPF	polypropylene fibers
CF	carbon fiber
SEM	Scanning electron microscopy
PE	Polyethylene
PVA	Polyvinyl alcohol
CNT	Carbon nanotube
CB	Carbon black
FCR	Fractional change in resistivity
